# Supplementary material for: Structural and regulatory determinants of flagellar motility in Rhodobacterales—the archetypal flagellum of Phaeobacter inhibens DSM 17395
Source: mSystems. 2025 Jul 8;10(8):e00419-25. doi: 10.1128/msystems.00419-25 (PMC12363192; doi:10.1128/msystems.00419-25)
Supplement: Figure S1 — Phylogenomic trees of Rhodobacterales and their flagella. [file msystems.00419-25-s0001.pdf]

Phylogenomic-Tree and Flagellar-Phylogeny of 306 Genome-sequenced *Rhodobacterales*

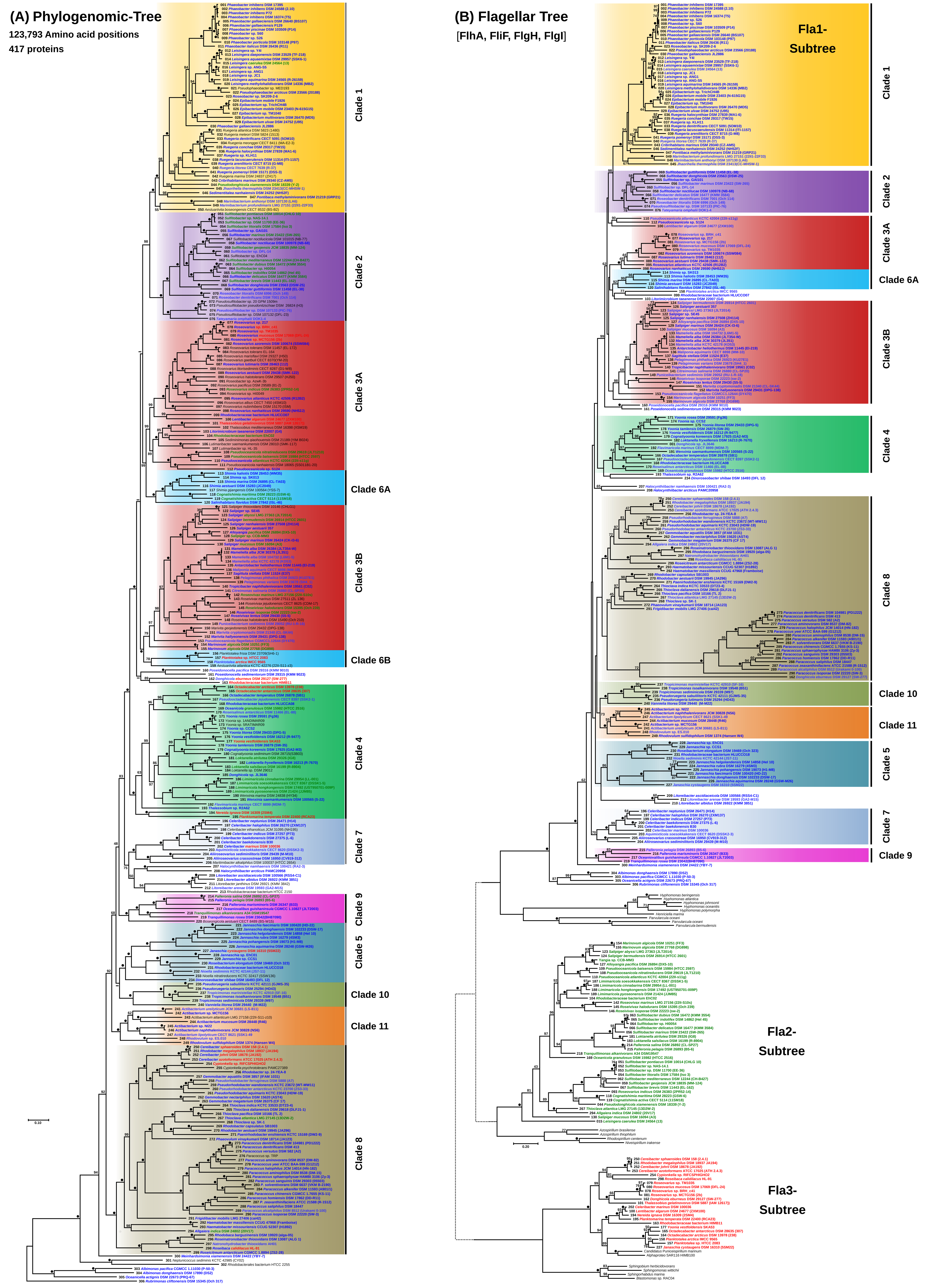

Supplementary Figure S1: (A) Phylogenomic RaxML tree of 306 *Rhodobacterales* based on 417 universal genes and 123,793 amino acid positions. Strains with 'Flagellar Gene Clusters' (FGCs) are highlighted in bold; the color code corresponds to the three FGC-types: *fla1*: blue, *fla2*: green, *fla3*: red. The presence of two colors reflects the presence of two FGCs, three FGCs were not found in any of the genomes examined (Table S1). Eleven different well-supported clades are color coded according to a former study of Bartling et al. (2018). **(B) Flagellar trees** of Fla1 (blue), Fla2 (green) and Fla3 (red) are based on alignments of four concatenated flagellar proteins (FlhA, FliF, FlgH, FlgI; Frank et al. 2015). The subtrees were individually rooted with flagellar systems from other closely related alphaproteobacterial orders. Subtrees in the Fla1 subtree that show a synchronous evolution to the phylogenomic tree are highlighted with the color code for the different clades used in Supplementary Figure S1A.
