# Supplementary material for: Structural and regulatory determinants of flagellar motility in Rhodobacterales—the archetypal flagellum of Phaeobacter inhibens DSM 17395
Source: mSystems. 2025 Jul 8;10(8):e00419-25. doi: 10.1128/msystems.00419-25 (PMC12363192; doi:10.1128/msystems.00419-25)
Supplement: Figure S2 — Subtrees for fla2 and fla3-type flagella. [file msystems.00419-25-s0002.pdf]

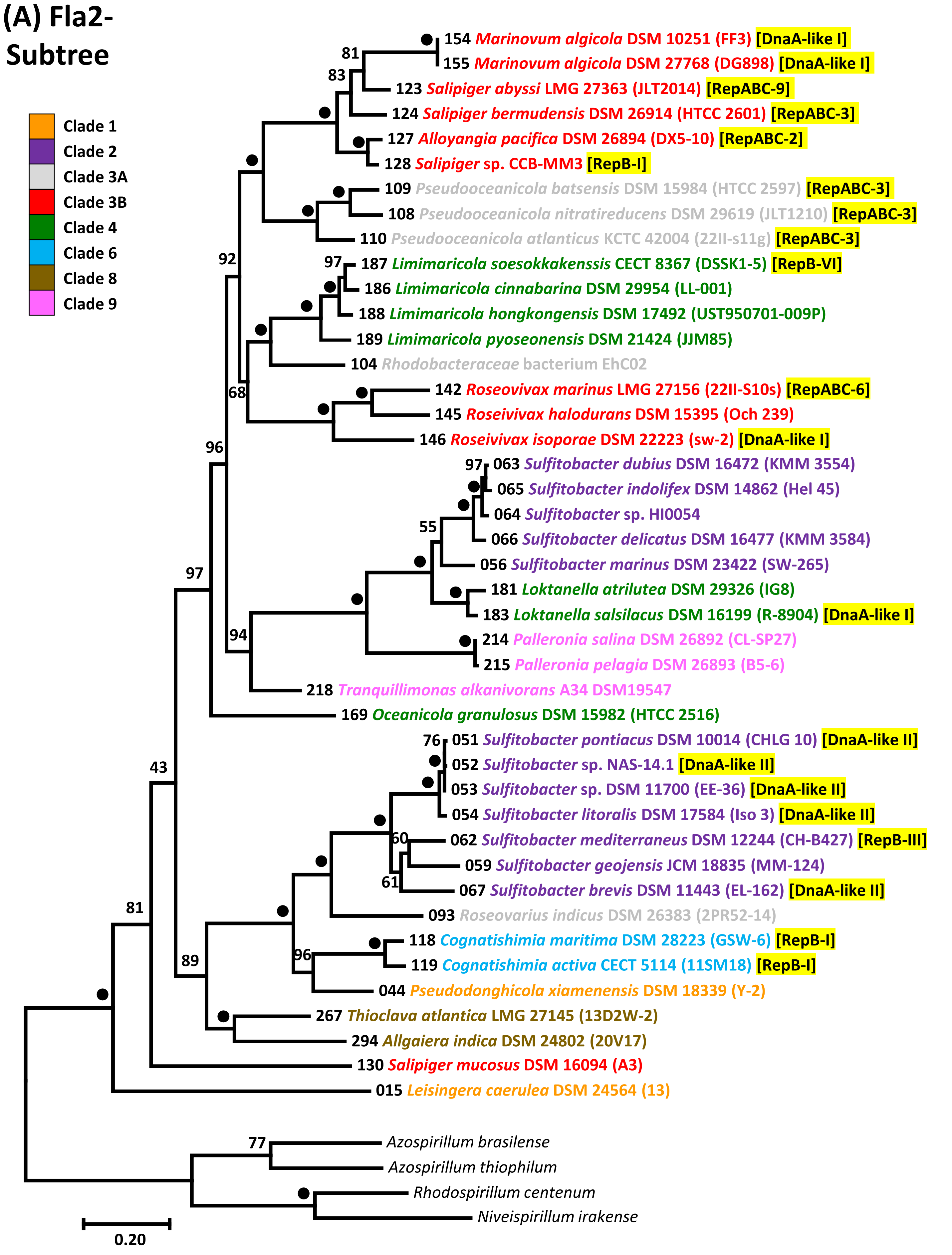
(A) Fla2-Subtree
Clade 1
Clade 2
Clade 3A
Clade 3B
Clade 4
Clade 6
Clade 8
Clade 9
154 Marinovum algicola DSM 10251 (FF3) [DnaA-like I]
155 Marinovum algicola DSM 27768 (DG898) [DnaA-like I]
123 Salipiger abyssi LMG 27363 (JLT2014) [RepABC-9]
124 Salipiger bermudensis DSM 26914 (HTCC 2601) [RepABC-3]
127 Alloyangia pacifica DSM 26894 (DX5-10) [RepABC-2]
128 Salipiger sp. CCB-MM3 [RepB-I]
109 Pseudooceanicola batsensis DSM 15984 (HTCC 2597) [RepABC-3]
108 Pseudooceanicola nitratireducens DSM 29619 (JLT1210) [RepABC-3]
110 Pseudooceanicola atlanticus KCTC 42004 (22II-s11g) [RepABC-3]
187 Limimaricola soesokkakensis CECT 8367 (DSSK1-5) [RepB-VI]
186 Limimaricola cinnabarina DSM 29954 (LL-001)
188 Limimaricola hongkongensis DSM 17492 (UST950701-009P)
189 Limimaricola pyoseonensis DSM 21424 (JJM85)
104 Rhodobacteraceae bacterium EhC02
142 Roseovivax marinus LMG 27156 (22II-S10s) [RepABC-6]
145 Roseivivax halodurans DSM 15395 (Och 239)
146 Roseivivax isoporae DSM 22223 (sw-2) [DnaA-like I]
063 Sulfitobacter dubius DSM 16472 (KMM 3554)
065 Sulfitobacter indolifex DSM 14862 (Hel 45)
064 Sulfitobacter sp. HI0054
066 Sulfitobacter delicatus DSM 16477 (KMM 3584)
056 Sulfitobacter marinus DSM 23422 (SW-265)
181 Loktanella atrilutea DSM 29326 (IG8)
183 Loktanella salsilacus DSM 16199 (R-8904) [DnaA-like I]
214 Palleronia salina DSM 26892 (CL-SP27)
215 Palleronia pelagia DSM 26893 (B5-6)
218 Tranquillimonas alkanivorans A34 DSM19547
169 Oceanicola granulosus DSM 15982 (HTCC 2516)
051 Sulfitobacter pontiacus DSM 10014 (CHLG 10) [DnaA-like II]
052 Sulfitobacter sp. NAS-14.1 [DnaA-like II]
053 Sulfitobacter sp. DSM 11700 (EE-36) [DnaA-like II]
054 Sulfitobacter litoralis DSM 17584 (Iso 3) [DnaA-like II]
062 Sulfitobacter mediterraneus DSM 12244 (CH-B427) [RepB-III]
059 Sulfitobacter geojensis JCM 18835 (MM-124)
067 Sulfitobacter brevis DSM 11443 (EL-162) [DnaA-like II]
093 Roseovarius indicus DSM 26383 (2PR52-14)
118 Cognatishimia maritima DSM 28223 (GSW-6) [RepB-I]
119 Cognatishimia activa CECT 5114 (11SM18) [RepB-I]
044 Pseudodonghicola xiamenensis DSM 18339 (Y-2)
267 Thioclava atlantica LMG 27145 (13D2W-2)
294 Allgaiera indica DSM 24802 (20V17)
130 Salipiger mucosus DSM 16094 (A3)
015 Leisingera caerulea DSM 24564 (13)
Azospirillum brasilense
Azospirillum thiophilum
Rhodospirillum centenum
Niveispirillum irakense
81
83
92
97
68
96
55
97
94
43
76
60
61
96
81
89
77
0.20

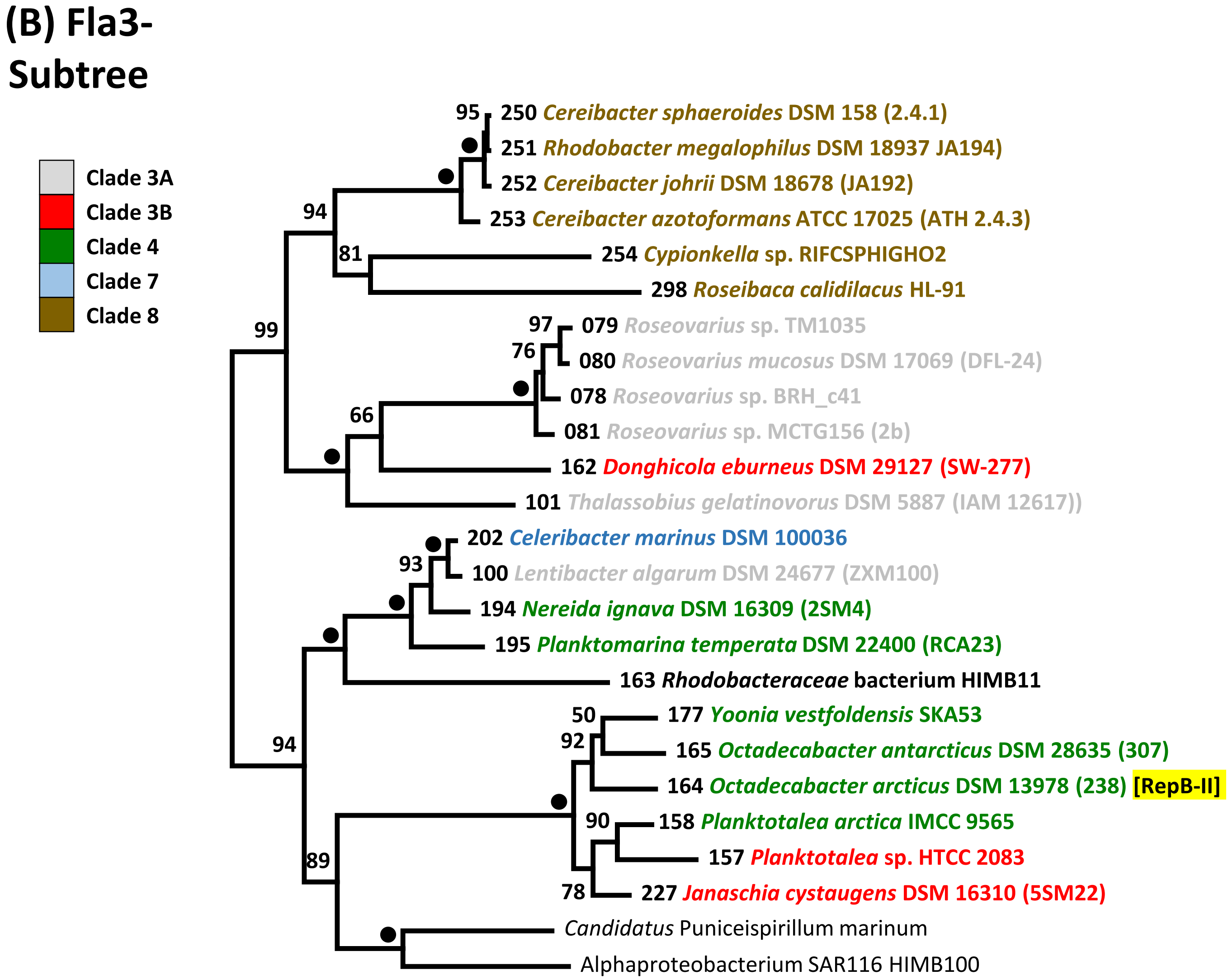

Supplementary Figure S2: Subtrees of fla2 and fla3 flagella systems. (A) Fla2 subtree (FlhA, FliF, FlgH, FlgI) corresponding to Figure S1B. *Rhodobacterales* taxa with *fla2*-type flagellar gene clusters were color-coded according to the different clades (Supplementary Figure S1A). The different plasmid compatibility groups are shown in brackets and highlighted in yellow. **(B) Fla3 subtree** (FlhA, FliF, FlgH, FlgI) corresponding to Supplementary Figure S1B. *Rhodobacterales* taxa with *fla3*-type flagellar gene clusters were color-coded according to the different clades (Supplementary Figure S1A). Plasmid compatibility groups are shown in brackets and highlighted in yellow.
