# Supplementary material for: Structural and regulatory determinants of flagellar motility in Rhodobacterales—the archetypal flagellum of Phaeobacter inhibens DSM 17395
Source: mSystems. 2025 Jul 8;10(8):e00419-25. doi: 10.1128/msystems.00419-25 (PMC12363192; doi:10.1128/msystems.00419-25)
Supplement: Figures S3 and S4 — Motility assays and electron microscopy of P. inhibens strains. [file msystems.00419-25-s0003.pdf]

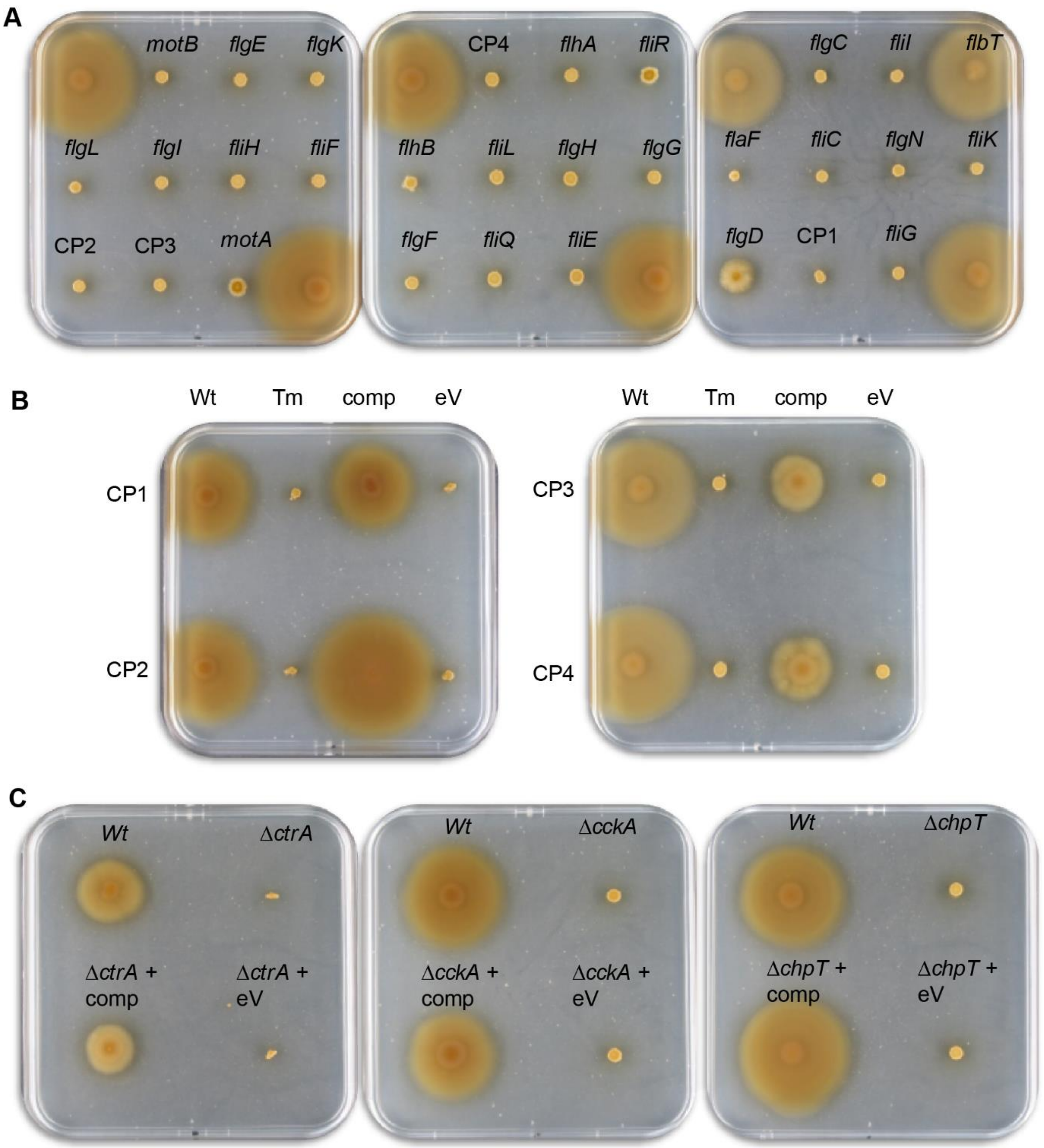

Supplementary Figure S3 – Motility assays for selected *P. inhibens* strains (A) Tn mutants in the FGC. *P. inhibens* wild type is used as a control on each plate. **(B) CP1-4 Tn mutants**, complements and controls. **(C) Tn mutants of CtrA phosphorelay genes**, complements and controls. Tm = transposon mutant, comp. = complementation with gene on plasmid, eV = empty vector control.

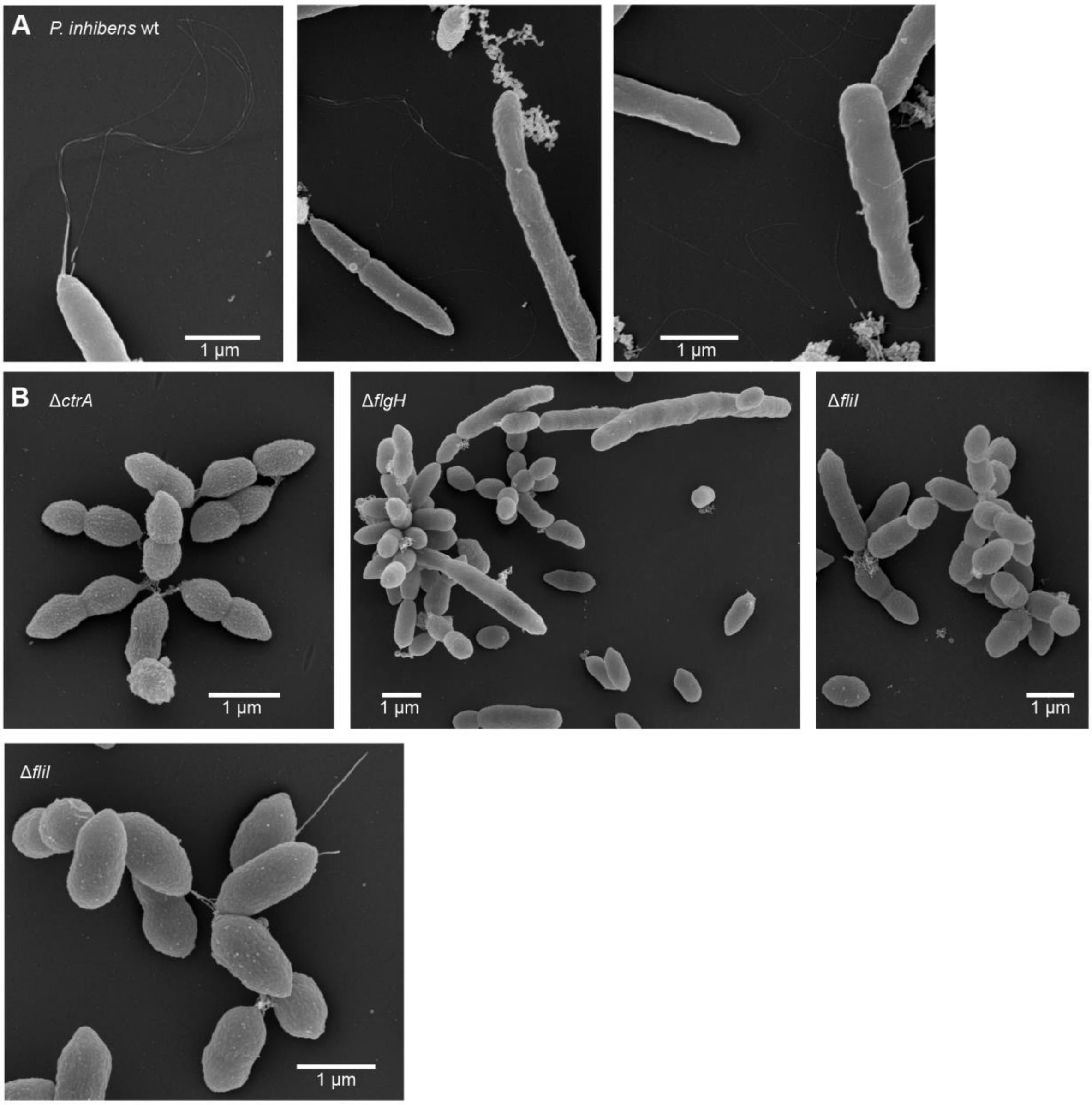

Supplementary Figure S4 – Electron microscopy of (A) *P. inhibens* wild type, showing flagella attached to cells and broken fragments, **(B)** transposon knockouts of motility genes.

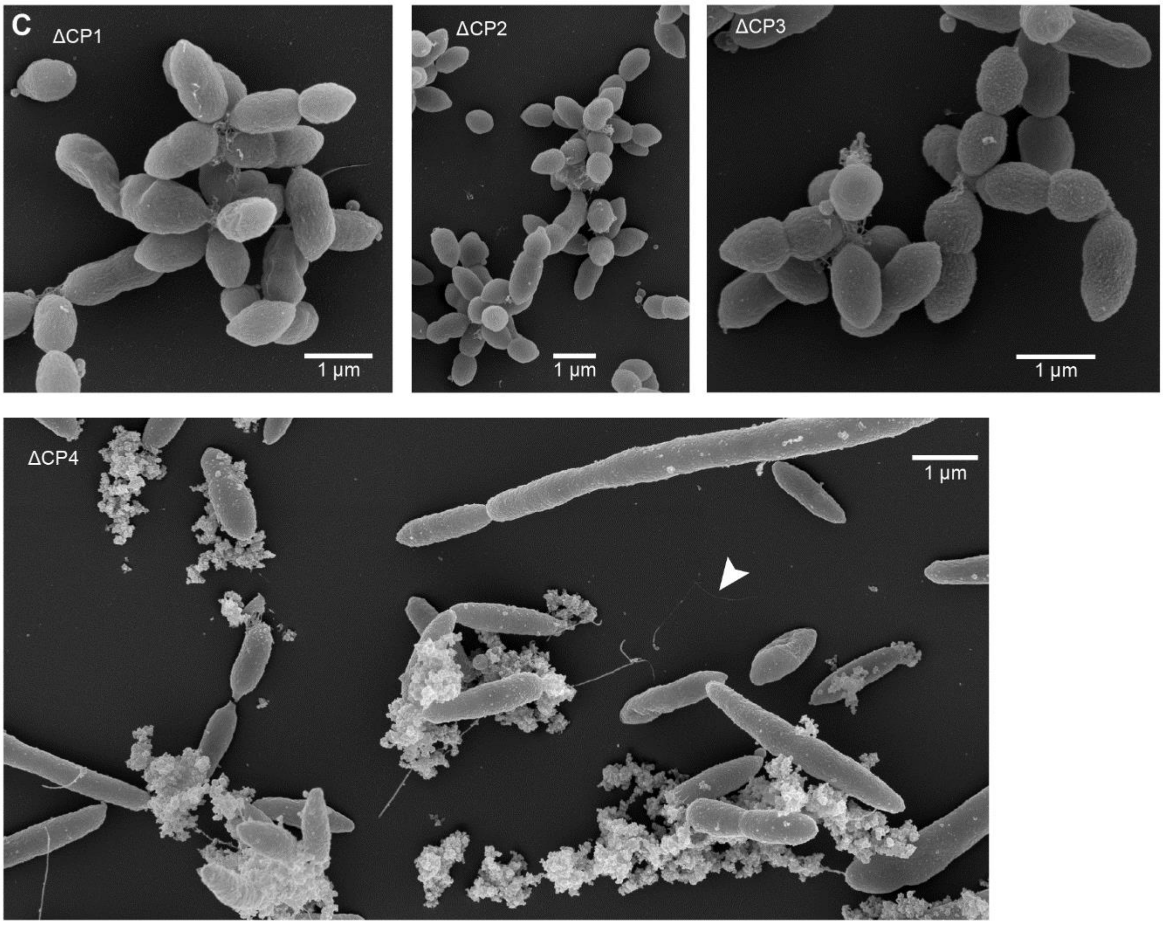

Supplementary Figure S4 – Electron microscopy of (C) *P. inhibens* transposon knockouts of CP1 to CP4.
